# Analysis of Narrow-Leaf Lupin Proteins in Lupin-Enriched Pasta by Untargeted and Targeted Mass Spectrometry

**DOI:** 10.3390/foods9081083

**Published:** 2020-08-08

**Authors:** Gilda Aiello, Yuchen Li, Giovanna Boschin, Marco Stanziale, Carmen Lammi, Anna Arnoldi

**Affiliations:** 1 Department of Human Science and Quality of Life Promotion, Telematic University San Raffaele, 00166 Rome, Italy; 2 Department of Pharmaceutical Sciences, University of Milan, 20133 Milan, Italy; yuchen.li@unimi.it (Y.L.); giovanna.boschin@unimi.it (G.B.); carmen.lammi@unimi.it (C.L.); anna.arnoldi@unimi.it (A.A.); 3 Department of Research and Development, Rustichella d’Abruzzo S.p.a., 65019 Pianella (PE), Italy; marco@rustichella.it

**Keywords:** food quality, liquid chromatography-tandem mass spectrometry LC-MS/MS, functional foods, lupin-enriched pasta, label free quantification, γ-conglutin, *Lupinus angustifolius*

## Abstract

The supplementation of different food items with grain legumes and, in particular, with lupin has been demonstrated to provide useful health benefits, especially in the area of cardiovascular disease prevention. In this work, label free quantitative untargeted and targeted approaches based on liquid chromatography−electrospray ionization−tandem mass spectrometry (LC−ESI−MS/MS) for investigating the protein profile of three pasta samples containing different percentages of narrow-leaf lupin flour were carried out. The untargeted method permitted the identification of the main acidic globulins (α-conglutin, β-conglutin, and δ-conglutin) and the comparison of their profile with raw lupin flour. The targeted method, based on High-performance liquid chromatography electrospray ionization tandem mass spectrometry HPLC-Chip-Multiple Reaction Monitoring (MRM) mode, allowed the quantification of γ-conglutin, the main hypoglycemic component of lupin protein: its concentration was around 2.25 mg/g in sample A, 2.16 mg/g in sample D, and 0.57 mg/g in sample F.

## 1. Introduction

The role of foods and food components in the prevention of several diseases is increasingly being acknowledged worldwide. For this reason, many food companies have plans for improving the nutraceutical properties of their products or developing innovative functional foods, especially targeting hypercholesterolemia, hypertension, diabetes, obesity, and metabolic syndrome. Furthermore, food industries are also constantly developing strategies to improve the nutritional quality of their products, particularly by incorporating low-glycemic index ingredients to address the demand for healthy products and to foster the particular niche of consumers with diabetes mellitus [1].

During the past 15 years, lupin flour has been increasingly used as a food ingredient because of its interesting nutritional and techno-functional properties [2]. Out of the four domesticated species, the commercially available raw materials derive from *Lupinus albus* (white lupin) and *Lupinus angustifolius* (narrow-leaf lupin). Due to its high protein content, various researchers have recently investigated the addition of lupin flour in a variety of cereal-based products, like cakes, pancakes, biscuits, or brioche [3], as well as spaghetti, pasta, and crisps [4]. In addition, lupin flour is also used as a protein-rich ingredient in gluten-free items because it does not contain any gluten [5].

Besides its useful nutritional features, numerous studies have shown that lupin provides interesting health benefits [6], particularly in the area of hyperglycemia control [7,8], hypertension prevention [9], and cholesterol reduction [10,11,12].

Protein seems to play a main beneficial role in these effects [13], a fact that points out the importance of the validation of adequate techniques for the assessment of the quality of the protein profile in processed food products. Although the technologies for pasta production are certainly well established, the enrichment of traditional pasta with other ingredients, such as lupin flour, may lead to an alteration of the quality of pasta, influencing the final composition as well as the texture and techno-functional properties, such as firmness, hardness, and cohesiveness. The need to establish food quality in terms of protein content after the addition of new ingredients is a fundamental prerequisite for food industries. On this basis, the first goal of this work was to optimize a proteomic approach based on mass spectrometry (MS) for evaluating the protein profile of some lupin-enriched pasta samples produced using different raw materials and food processing conditions. Shotgun proteomics, which is currently considered a fast and effective screening method, was selected for the characterization of these products. In details, based on the intensity-based absolute quantification (iBAQ), the changes of soluble lupin proteins among lupin-enriched pasta in comparison to raw materials were explored. Despite the fact that some MS-methods have already been developed for detecting and quantifying the main storage proteins of white lupin in beverages and protein isolates [14], as well as in pasta and biscuits using rapid shotgun proteomics methods [15], literature does not report any methodology for the detection of narrow-leaf lupin protein in the final products. In addition, there are no reports aimed at evaluating how the lupin protein profile is modified by food processing techniques. In order to overcome this limit, the untargeted method based on high pressure liquid chromatography-tandem mass spectrometry (HPLC-MS/MS) analysis, implemented by software-based data mining and complementary bioinformatics evaluation, as a tool for the rapid detection of the total profile of narrow-leaf lupin proteins in some lupin-enriched pasta samples was presented. The selectivity and the specificity of shotgun proteomics, however, was implemented by the optimization of a method for the quantitative measurement of narrow-leaf lupin γ-conglutin in pasta based on multiple reactions monitoring (MRM). When compared to white bread, the consumption of bread enriched with narrow-leaf lupin flour reduces the post-prandial glucose response in healthy adults, mediated by an increased insulin response [16]. Among the lupin proteins, **γ**-conglutin certainly has a crucial role in this activity since pasta supplemented with isolated **γ**-conglutin reduces plasma glucose concentration in rats after glucose overload trial [17] as well as in humans [18]. Having specific and selective analytical tools aimed at quantifying even low levels of proteins such as **γ**-conglutin in lupin-enriched foods, as well as for the assessment of its integrity when it is incorporated into complex food matrices is an increasingly strong demand from industries. The only methods described in literature have been developed for the analysis of white lupin **γ**-conglutin in lupin protein extracts [19] or in pasta and biscuits [15]. In spite of this interest with *L. albus*, little is known about the **γ**-conglutin from narrow-leaf lupin kernel. Moreover, the complexity of the **γ**-conglutin in the range of lupin species in terms of gene structure, phylogenetic relationships, and the protein abundance during seed development support the MRM-method development for **γ**-conglutin quantification. The accurate quantification of multiple target proteins in a very complex mixture, such as pasta, is a challenging issue that requires highly sensitive and high-throughput assays like those provided by MRM analysis [20,21].

## 2. Materials and Methods 

### 2.1. Chemicals, Enzymes, and Solvents

All chemicals and reagents were of analytical grade. LC-grade H_2_O (18 MΩ cm) was prepared with a Milli-Q H_2_O purification system (Millipore, Bedford, MA, USA). Acetonitrile (ACN), tris(hydroxymethyl)aminomethane (Tris-HCl), hydrochloric acid (HCl), ammonium bicarbonate, 2-iodoacetamide (IAM), 1,4-dithiothreitol (DTT), trypsin from bovine pancreas (T1426, lyophilized powder, ≥10,000 units/mg protein), and pepsin from porcine gastric mucosa (P7012, lyophilized powder, ≥2500 units/mg protein) were from Sigma-Aldrich (St. Louis, MO, USA). Bovine serum albumin (BSA), Mini-Protean apparatus, precision plus protein standards, Bradford reagent, and Coomassie Blue G-250 were purchased from Bio-Rad (Hercules, CA, USA).

### 2.2. Analyzed Samples

All samples were provided by the company Rustichella d’Abruzzo (Pianella (PE), Italy), an artisanal pasta factory. They were unprocessed raw materials (flours), i.e., narrow-leaf lupin kernel flour (LK), durum wheat flour (DW), Mix (consisting of 58% of lupine flour, 33% of durum wheat, 5% of atomized lemon juice, 2% of vegetal fiber, and 2% of carob flour), as well as three samples of lupin enriched pasta products (samples A, D, and F). All pasta products were produced by adding lupin ingredients to DW in order to ensure a desired texture and cooking quality for the products. Specifically, sample A was constituted of 56% of LK and 44% of DW; sample D was constituted of 58% of LK and 42% of DW. Sample F was made of 100% Mix. During the processing phase, the temperature of the water used to knead the dough of samples A and D was 28 °C, while for sample F, the temperature was 38 °C. Pasta samples A and D were then dried for about 16 h at 52 °C, whereas pasta sample F was dried for 16 h at 56 °C.

### 2.3. Protein Extraction and Sodium Dodecyl Sulphate-Ppolyacrylamide Gel Electrophoresis (SDS-PAGE)

The raw materials were extracted as such, whereas sample A, D, and F were previously manually ground to obtain a fine and homogeneous powder. To remove lipids, 5 g of each sample was defatted with 100 mL of hexane overnight under magnetic stirring. After drying at room temperature, 1 g of defatted material was suspended in 10 mL of Tris-HCl 100 mM/NaCl buffer (0.5 M, pH 8.2) and the protein extraction was carried out at 60 °C for 6 h under magnetic stirring. The solid residue was eliminated by centrifugation at 5800× *g* at 4 °C for 30 min and the supernatant was dialyzed against 100 mM Tris-HCl buffer, pH 8.0, at 4 °C for 36 h. The protein concentration was determined by the colorimetric Bradford. Analyses were carried out at a wavelength λ = 595 nm. To determine protein concentration, a standard curve based on BSA was employed. The efficiency of protein extraction was evaluated by SDS-PAGE. Then, 12% of polyacrylamide was used with Tris–glycine buffer (pH 8.3, 0.1% SDS).

### 2.4. Tryptic and Peptic Digestions

In order to improve the efficiency of the enzymatic digestion, the samples were previously heated at 60 °C for 45 min to unfold the protein structure. The reduction and alkylation processed was similar to those reported in [15]. Two different enzymatic digestions were employed. The tryptic digestion, used for untargeted MS analysis, was performed by adding 5 μL of 2 mg/mL trypsin solution to 100 μL of purified protein extract (protein/trypsin ratio of 50:1) and carried out at 37 °C overnight. The digestion reaction was stopped with 1% formic acid. In parallel, the peptic digestion was employed to hydrolyze the γ-conglutin that was subsequently quantified by MRM-MS. The peptic digestion was performed by changing the pH of the protein extract to 2 by adding 0.1 M HCl. Then, 5 μL of 2 mg/mL pepsin were added to 100 μL of the purified protein extract (protein/trypsin ratio of 50:1) and carried out at 37 °C overnight. The digestion reaction was quenched by changing the pH to neutral.

### 2.5. Untargeted Shotgun Analysis by LC-ESI-MS/MS and Data Processing

Digested samples were purified using SepPak C18 cartridges (Thermo Fisher Scientific, Life Technology, Milan, Italy), dried in a Speed-Vac (Martin Christ, Germany) and then reconstituted with 50 μL of a solution of 98% water and 2% ACN containing 0.1% formic acid. Aliquots of 5 µL of tryptic peptides were injected in a nano-chromatographic system, HPLC-Chip (Agilent Palo Alto, CA, USA). The analysis was conducted on a SL IT mass spectrometer. LC-MS/MS analysis was performed in data-dependent acquisition AutoMS (n) mode. In order to increase the number of identified peptides, three technical replicates (LC-MS/MS runs) were run for each hydrolysate. Both the detailed chromatographic separation conditions and the MS parameters used are reported in the Supplementary Materials. PEAKS Studio 8.5 software (Bioinformatics Solutions Inc., Waterloo, ON, Canada) was used for the automated peptide identification from tandem mass spectrogram. The *Viridiplantae* protein sequences database downloaded from SwissProt Uniprot (2018) was consulted. Carbamidomethylation was chosen as a fixed modification. Parent mass and fragment mass error tolerance were set at 1.2 and 0.8 Da, respectively. Protein confidence levels were set to a 1% false discovery rate (FDR) and at least ≥2 peptide/protein with 1% FDR at the peptide level were used to filter out inaccurate proteins for the PEAKS search. “De novo only” analysis was also included in the search. A −10lgP > 20 indicated that the detected proteins were relatively high in confidence, as it targeted very few decoy matches above that threshold. The hierarchical clustering analysis (HCA) and its visualization were performed using Cluster 3.0 and Java TreeView, respectively. The complete linkage method was then used in the assignment of clusters.

### 2.6. Label Free Quantification

Protein quantification was established on the intensity-based absolute quantification (iBAQ) method provided by Scaffold [22]. Protein intensities were provided by PEAKs as the sum of all the identified peptide intensities per protein. The iBAQ values were obtained by dividing the protein intensities by the number of theoretically observable peptides of each protein (calculated by in silico protein digestion). The tryptic peptides taken in account had lengths between 6 and 30 amino acids [23,24]. The average of iBAQ values of the three technical replicates for each protein was considered as the “protein abundance”, providing an accurate determination of the relative abundance of all proteins identified in each sample.

### 2.7. MRM Method Optimization and Validation for the γ-Conglutin Quantification

The quantification of γ-conglutin was performed by analyzing the peptic digests through multiple reaction monitoring (MRM) mass spectrometry, monitoring two diagnostic peptides, PNNIQ (control peptide) and NIHKRTPL (quantification peptide), both belonging univocally to γ-conglutin. Two transitions were monitored for each peptide as reported in the “Results” section. Both peptides were eluted applying the following gradient: 20% solvent B (0 min), 95% solvent B (0–30 min), and back to 5% in 5 min. The drying gas temperature was set at 300 °C, flow rate 3 L/min (nitrogen). Data acquisition was carried out in positive ionization mode. Capillary voltage was −1970 V, with endplate offset −500 V. Full scan mass spectra were acquired in the mass range from *m/z* 300 to 2000 Da. Three technical replicates (LC–MS/MS runs) were run for each hydrolysate. Analytical parameters, i.e., LOQ and LOD, were measured to ensure appropriate performance of the developed method. The accuracy of the assay was assessed, spiking DW flour with the standard peptide PNNIQ at 25 μg/mL. The sensitivity of the method was calculated by the LOQ’s [signal-to-noise (S/N) = 10] and LOD’s (S/N = 3). The analytical validation study evaluated the assay accuracy, precision (intra- and inter-day repeatability), linearity, and recovery. 

### 2.8. Statistical Analysis

From a statistical point of view, both the extraction of proteins from each sample and the protein digestion were performed three times. The peptides obtained from each independent digestion (i.e., tryptic and peptic) were then pooled and analyzed by MS with three technical replicates (n = 3), respectively, and the results are represented as mean ± SD unless otherwise mentioned.

## 3. Results

### 3.1. Qualitative Comparison of the Protein Profile of the Pasta Samples by SDS-PAGE and MS Analysis

Acidic globulins and albumins, in respect to wheat prolamins and glutelins, were extracted from the raw materials and pasta samples by using an alkaline buffer containing NaCl. The concentration of the soluble protein in each sample was assessed by the Bradford method. The soluble protein content of the starting materials was as follows: DW 4.57 ± 0.5 μg/μL, LK 5.05 ± 0.2 μg/μL, and Mix 0.78 ± 0.2 μg/μL. The low value of the last sample probably depended on an inefficient extraction, possibly caused by the presence of the carob flour and vegetal fiber that hindered the protein extraction. The protein contents of the pasta samples A, D, and F were equal to 3.5 ± 0.3, 3.8 ± 0.4, and 2.7 ± 0.3 μg/μL, respectively, indicating that samples A and D have similar protein contents, whereas sample F has a much lower protein content, possibly linked to the inclusion of Mix in its formulation.

**Figure 1:**
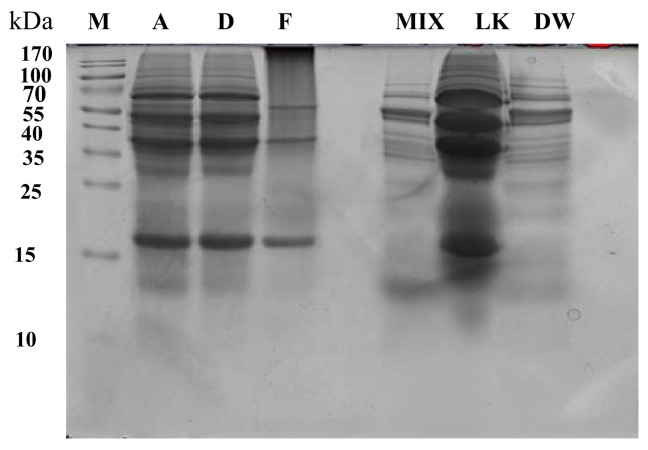
Reduced SDS-PAGE protein profile of lupin-enriched pasta samples and raw materials; M, pre-stained molecular marker; A, D, and F lupin-based pasta samples; Mix, LK: lupin kernel, and DW: durum wheat. Each sample (10 μL) was added to 10 μL of loading buffer, loading 20 μL for each well.

Aiming to deeply investigate the differences in protein composition among the lupin-enriched pasta samples and the raw materials, a proteomic approach based on SDS-PAGE, after sulfur bridges reduction, was selected as a tool for a visual screening of the molecular weight protein distribution among the samples. The results of this analysis are shown in Figure 1. Starting from the raw materials, the samples Mix and DW showed very similar protein bands due to the high prevalence of wheat proteins, whereas LK had a completely different pattern, where lupin legumins and vicilins are easily recognized [8]. In fact, the protein profile of narrow-leaf lupin is characterized by the presence of proteins with molecular weights falling in the range of 68 to 15 kDa [25]. The intense bands around 53–74 kDa indicate the presence of the acid and basic subunits of α-conglutin (legumins or 11S globulins). On the contrary, β-conglutin (vicilins or 7S globulins), which does not contain any disulfide bridge, is a very heterogeneous protein fraction that produces numerous bands falling in the range 15–72 kDa [26]. Finally, the intense band at 16 kDa may be attributed to the small subunit of γ-conglutin [25]. Considering now the pasta samples, lupin proteins are clearly visible in the SDS-PAGE’s of A and D, which are very similar, whereas the profile of sample F is very different, with the absence of α-conglutin and the low presence of β-conglutin.

To get a deeper characterization of the protein composition of each sample, a shotgun proteomic approach was adopted, since comparative proteomics currently represents the most up-to-date method for characterizing proteome changes consequent to food processing [27]. The peptide obtained by tryptic digestion was analyzed by nano-electrospray ionization tandem mass spectrometry nESI-LC-MS/MS. Data obtained by Data-Dependent-Acquisition (DDA) mode were analyzed to identify lupin tryptic peptides assessing the sequence coverage for each conglutin. In particular, by comparing MS/MS data against Viridiplantae database, α, β, and δ-conglutin from *L. angustifolius* were easily identified (several isoforms were detected for each protein), whereas no peptides belonging to γ-conglutin could be found. This phenomenon may be explained by the well-known resistance of γ-conglutin to tryptic hydrolysis [28]. Besides proteins belonging to the Lupinus genus, proteins belonging to different phylogenetic species were identified, including monogalactosidase glycerol synthase 2 type B; uncharacterized protein (*Oryza sativa* subsp. Japonica); protein synthesis inhibitor I (*Hordeum vulgare*); Rho GTPase-activating protein 5 (*Arabidopsis thaliana*); legumin (*Phaseolus vulgaris*); and auxin response factor (*Spinacea oleracea*). Table S1 reports the peptides and proteins identified in each analyzed sample.

**Figure 2:**
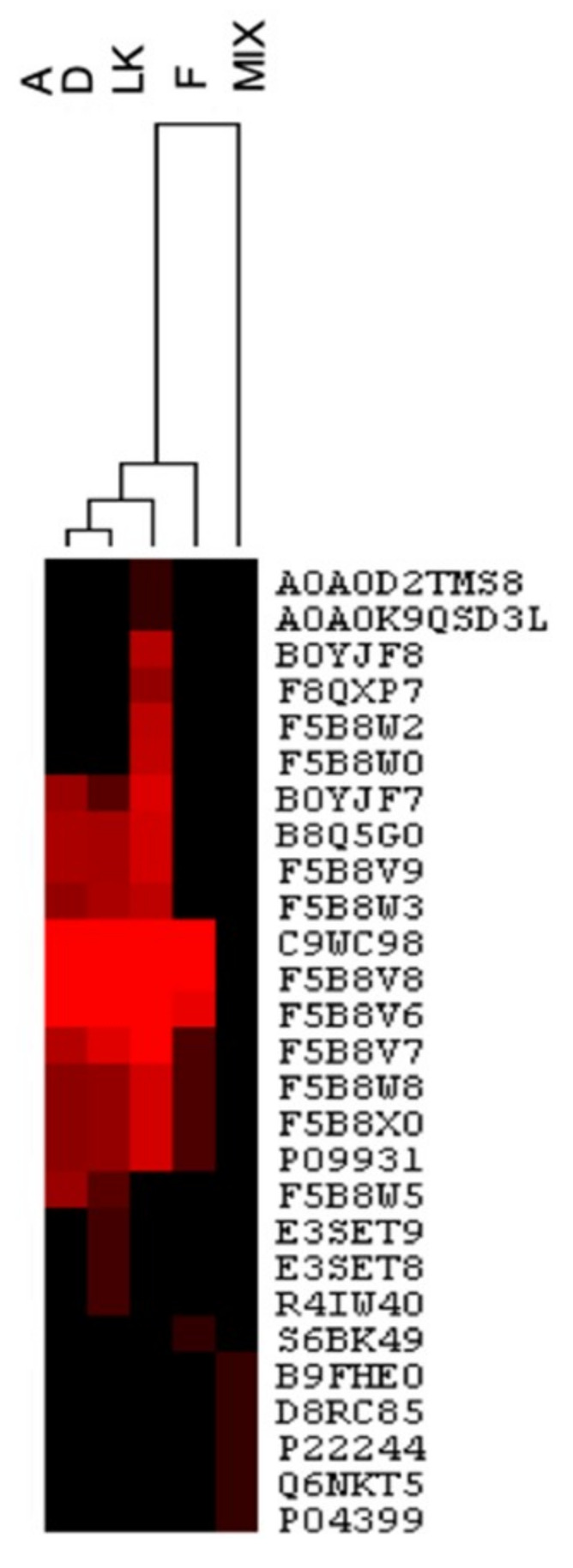
Hierarchical clustering analysis (HCA) with the dendrogram of the identified protein in lupin-based pasta samples and raw materials. Individual proteins are given in columns. Heat map colors are bases on values of −10LgP (protein confidence score), combined with hierarchical clustering of samples.

The subsequent quantitative evaluation was focused only on the proteins belonging to *L. angustifolius*. With the aim of highlighting the similarities between the pasta samples and the raw materials, it was decided to use HCA, which allows the presentation of cluster results in a dendrogram, where the similarity among the samples is shown by a smaller distance at which they join in a single cluster. By plotting −10LgP for each identified protein across the samples, the heatmap reported in Figure 2 clearly shows the correlation in terms of composition existing between sample A and D, which form a very narrow cluster. Both are then correlated with LK, whereas sample F and Mix fall at a wider distance, indicating a very different protein composition in respect to LK. 

### 3.2. Quantitative Comparison of Lupin-Based Pasta Samples and Raw Materials by an MS Label-Free Method

Trustworthy quantitative methods to determine the total protein content of foods and food ingredients are widely used, not only to guarantee the quality and safety of food, but also to get a comprehensive view of how technological processes change the protein profile of treated foods. Common processing-induced changes include variations in molecular weight distribution following hydrolysis, racemization, and/or oxidation of amino acids and protein cross-linking [29]. For example, label-free quantification methods have been widely used to highlight the differences in the peptide profile of Spanish Teruel, Italian, and Belgian dry-cured ham [30].

With the aim to get a relative quantification of lupin proteins in each pasta sample, an MS label-free method was adopted for protein quantification. The protein abundance was calculated by applying the iBAQ methodology, which has been described to have a good correlation with known relative protein amounts over at least four orders of magnitude [22]. It estimates protein abundance as the sum of intensities of all tryptic peptides identified for each protein divided by the theoretically observable peptides, obtained by in silico digestion, taking into account only peptides consisting of 6–30 amino acid residues. In this work, the resulting iBAQ intensities were used to provide an accurate determination of the relative abundance of all identified proteins.

**Figure 3:**
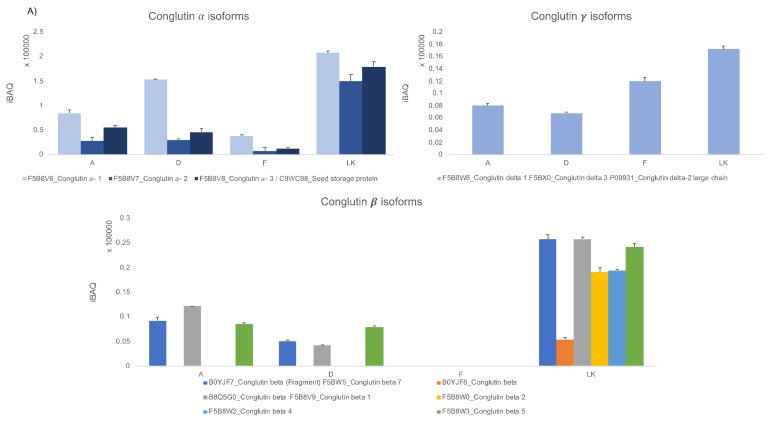

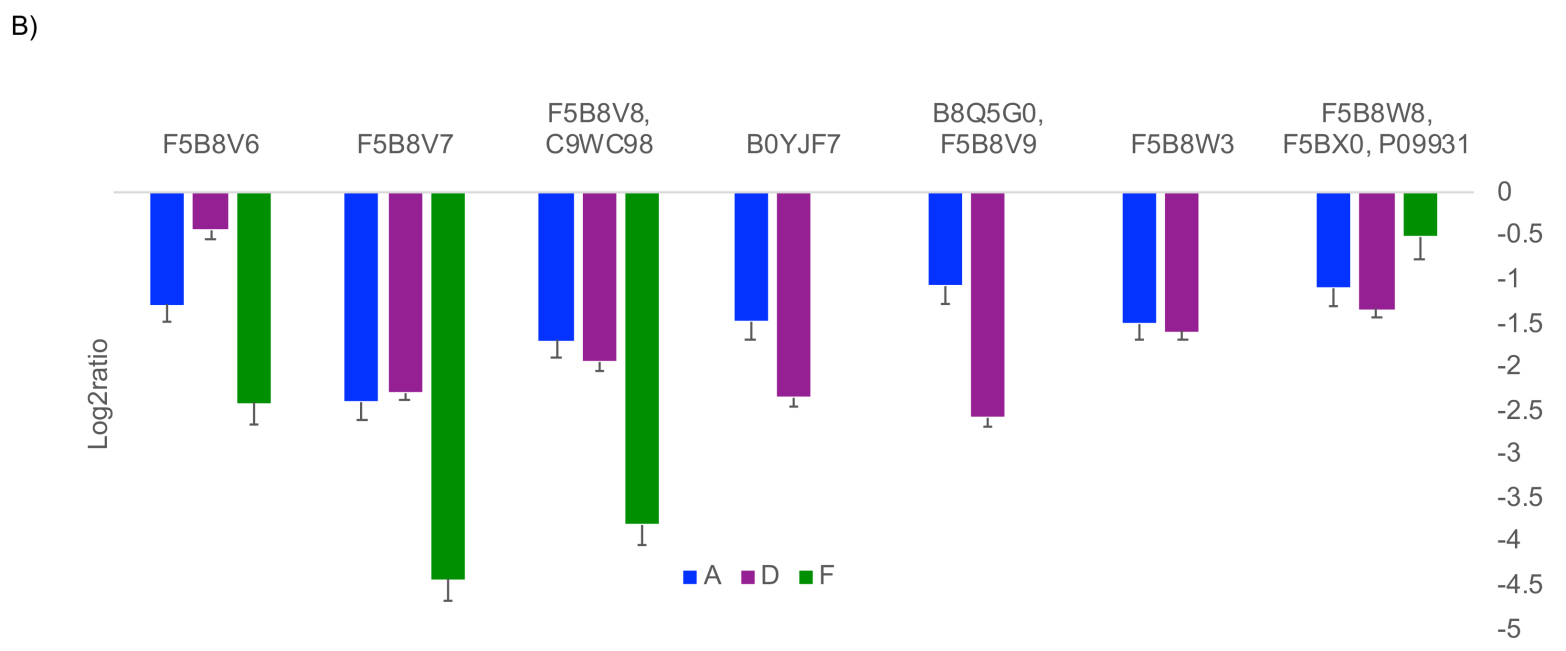
(**A**) iBAQ values of α, β, and δ-conglutin isoforms in samples A, D, F, and LK. (**B**) The log2 fold-changes of the protein levels in A, D, and F versus raw lupin flour (LK).

Most of the selected proteins were present at higher concentrations in the raw material LK, whereas they decreased in the processed samples, as reported in Figure 3A, which shows the trend of α, β, and δ-conglutin isoforms, respectively, in lupin-enriched pasta samples and LK. The three graphs show substantial protein differences among the samples. In order to highlight the variation in the relative abundance of each identified protein between processed lupin-enriched pasta and raw LK, the Log2 ratio was plotted as reported in Figure 3B. The higher the observed value of Log2 ratio, the higher the protein yields in the samples. Table 1 reports the relative percentages of each identified protein in pasta samples in comparison to those detected in raw LK. Specifically, conglutin α1 (F5B8V6) was detected as the most abundant protein in sample D, characterized by a 74% content in respect to LK, whereas the highest reduction in concentration was observed for conglutin α-2 (F5B8V7) in sample F, where its yield slumps to 4.63% versus LK (Figure 3B). On the contrary, sample F was characterized by the highest content of conglutin δ-2 large chain (F5B8W8), corresponding to 69% (Figure 3B). Overall, it is possible to affirm that A and D are the richest in lupin proteins, i.e., 11S α-conglutin and 7S β-conglutin, whereas F is the richest in the conglutin δ-2 large chain. The pasta samples described here have a very high lupin protein content in respect to literature where all the formulations comprise lupin ingredient percentages ranging from <5% to 30% [31,32,33]. The iBAQ strategy thus permits one to calculate the relative percentages of proteins in the samples. Here, it was used to monitor the impact of the different raw materials and processing conditions on the final protein composition. The rapidity of the untargeted method represents the main advantage in respect to the methods available in the literature which make use of the more elaborated MRM approach, even to detect abundant proteins that are easily identifiable with untargeted MS [15].

### 3.3. Development of a Targeted MRM-Assay for the Absolute Quantification of γ-Conglutin

Since the untargeted MS method used for profiling the differences among processed samples and raw ingredients did not provide any information about γ-conglutin, a specific targeted MRM assay was developed. It is well known that the γ-conglutin protein fraction of *L. angustifolius* is highly resistant to tryptic hydrolysis. Its insensitivity has been explained in [34]. However, the quantification of this protein in lupin-enriched food items is fundamental, since it is one of the main hypoglycemic lupin components. In order to increase the accuracy of γ-conglutin determination, it was decided to develop a sensitive MRM assay that is widely adopted for monitoring peptide integrity in nutritional investigations [35].

**Figure 4:**
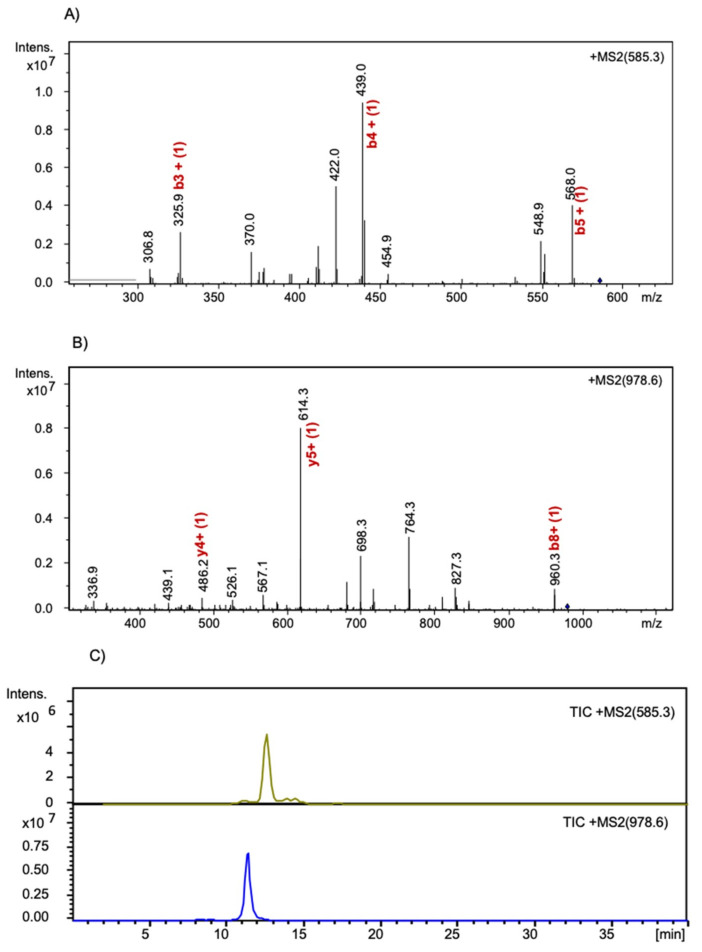
Electrospray ionization-tandem mass spectrometry spectra of [M + H]^+^ ions of (**A**) PNNIQ and (**B**) NIHKRTPL, respectively; (**C**) Total ion chromatograms (TIC) of PNNIQ (*m/z* 585.23) and NIHKRTPL (*m/z* 978.6).

The development consisted of three main steps: (1) selection of proteotypic peptides through in silico pepsin hydrolysis by PeptideCutter of γ-conglutin; (2) hydrolysis of the extracted proteins with pepsin; (3) method refinement by analyzing peptic hydrolysates of crude proteins extracted from pasta. In order to guarantee the specificity of the method, two peptides, i.e., PNNIQ and NIHKRTPL belonging to γ-conglutin 2 (F5B8W7, CONG2_LUPAN_SwissProt_Uniprot), were selected as proteotypic peptides to be monitored. Both met the stringent criteria required for this technique, i.e., good ESI ionization and no post-translational modifications, and 0 missed cleavage [36]. The adopted MS/MS strategy was suitable for the unambiguous determination and quantification of γ-conglutin in the complex pasta matrix, since the selected peptides belong univocally to *L. angustifolius* γ-conglutin. The absence of the selected prototypic peptide signals in a real food matrix was checked by analyzing DW (data not shown). The specificity and uniqueness of the selected marker peptides were verified by blasting, comparing the peptide sequences against the online accessible protein databases (SwissProt, Uniprot). With the aim to ensure greater specificity, the LC-MRM analysis was performed, monitoring two diagnostic transitions for each peptide. In order to obtain highly sensitive measurements, robust MS/MS transitions with high specificity and intensity for each peptide were monitored. The MRM transitions for PNNIQ were those from the mono-charged precursor ion [M + H]^+^ (*m/z* 585.3) to product-ions b4 and b5 with *m/z* 439.0 and 568.2, respectively. The MRM transitions for NIHKRTPL were from the mono-charged precursor ion [M+H]^+^ (*m/z* 978.6) to product-ions y5 and b8 with *m/z* 614.3 and 960.6, respectively, as shown in Figure 4. The peak areas of all monitored transitions from parent to product ions of NIHKRTPL were integrated and used for the quantification.

### 3.4. Validation of the Analytical Parameters: Range of Linearity, Sensitivity, LOQ, and LOD of γ-Conglutin Quantification in Lupin Based-Pasta

The linearity of the method was assessed in the concentration range, spanning three different orders of magnitude. Therefore, six different concentrations of standard peptide NIHKRTPL ranging from 25, 50, 250, 500, 1000, 2000, and 4000 μg/mL were analyzed in three replicates. To determine the relation between the peak area under the curves and the concentration of peptides, the calibration curve was built by plotting the mean response factor (peak area) against the respective concentrations of NIHKRTPL. The linearity of the method was determined by the correlation coefficient. A correlation coefficient greater than 0.95 and an intercept not significantly different from zero was accepted as criteria for a good standard regression curve. All peak area values were linear (R^2^ > 0.99) and the RDS% values were in all cases under 12%, thus showing good repeatability of the measurement. The accuracy of MRM assay was verified by adding known quantities of NIHKRTPL to the blank matrix (DW) at 250 μg/mL. The accuracy was detected as higher than 95%. LOQ was 0.25 mg/g, whereas LOD was detected equal to 0.20 mg/g.

**Figure 5:**
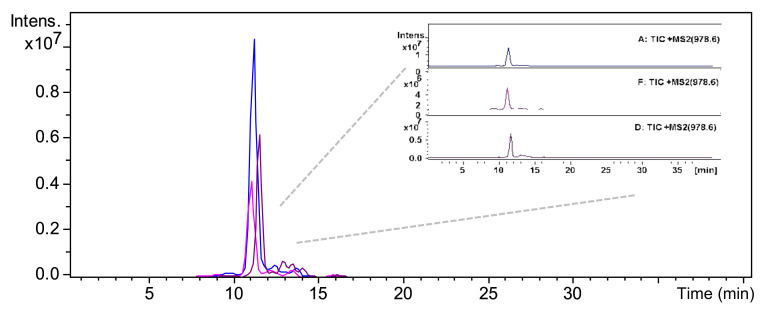
Extracted ion chromatograms of NIHKRTPL after mass spectrometry (MS) analysis in A, D, and F pasta samples.

The developed approach was then applied to the lupin-enriched pasta samples. Exemplary extracted ion chromatograms of NIHKRTPL in A, D, and F samples are shown in Figure 5. The amounts of γ-conglutin detected in A, D, and F samples, obtained by interpolating the signal of the targeted peptide over the calibration curve, are reported in Table 2. The highest γ-conglutin concentration was found in pasta sample A, containing 2.25 mg/g of γ-conglutin/pasta, followed by samples D (2.16 mg/g), and F (0.57 mg/g). Again, the fibers of samples F impaired the γ-conglutin detection. These results are in line with those reported in other commercial food products [15].

## 4. Conclusions

To the best of our knowledge, this is the first study on the effect of narrow-leaf lupin flour supplementation on the quality of pasta proteins. These integrated methods, based on either untargeted or targeted approaches, have a high potential for exploitation for the simultaneous detection of additional protein sources in various foods. The untargeted LC-MS/MS procedure enabled the estimation of the relative changes in protein abundance in all the examined samples. In addition, the sensitivity and selectivity obtained by the targeted proteomic-based MRM-LC-ESI-MS/MS approach allowed us to propose a useful quantitative method for the detection of a specific nutraceutical protein fraction in pasta supplemented with narrow-leaf lupin flour. Both the untargeted and targeted methods were demonstrated to be useful tools for investigating protein composition in lupin supplemented pasta. Finally, it is important to underline that the described pasta samples contain very large amounts of lupin protein, which has never been described before in literature.

## Figures and Tables

**Table 1:** iBAQ values and relative percentage of lupin protein identified in lupin-enriched pasta.

Protein Name Accession N.	A ± SD (iBAQ)	D ± SD (iBAQ)	F ± SD (iBAQ)	LK ± SD (iBAQ)	%A	%D	%F
Conglutin α-1_F5B8V6_LUPAN	(8.45 ± 0.74) × 10^4^	(1.53 ± 0.18) × 10^5^	(3.88 ± 0.14) × 10^4^	(2.07 ± 0.04) × 10^5^	40.82	73.91	18.74
Conglutin α-2_F5B8V7_LUPAN	(2.83 ± 0.73) × 10^4^	(3.05 ± 0.22) × 10^4^	(6.94 ± 0.74) × 10^3^	(1.50 ± 0.11) × 10^5^	18.87	20.33	4.63
Conglutin α-3_F5B8V8_LUPAN	(5.52 ± 0.52) × 10^4^	(4.66 ± 0.73) × 10^4^	(1.28 ± 0.26) × 10^4^	(1.79 ± 0.11) × 10^5^	30.84	26.03	7.15
Conglutin β _(Fragment)__B0YJF7_LUPAN	(9.18 ± 0.66) × 10^3^	(5.04 ± 0.24) × 10^3^		(2.57 ± 0.09) × 10^4^	35.72	19.61	
Conglutin β-1_F5B8V9_LUPAN	(1.22 ± 0.54) × 10^4^	(4.31 ± 0.43) × 10^3^		(2.58 ± 0.46) × 10^4^	47.29	16.71	
Conglutin β-2_F5B8W0_LUPAN				(1.90 ± 0.09) × 10^4^			
Conglutin β-4_F5B8W2_LUPAN				(1.94 ± 0.02) × 10^4^			
Conglutin β-5_F5B8W3_LUPAN	(8.47 ± 0.35) × 10^3^	(7.92 ± 0.23) × 10^3^		(2.41 ± 0.08) × 10^4^	35.15	32.86	
Conglutin β-_B0YJF8_LUPAN				(5.38 ± 0.34) × 10^3^			
Conglutin δ-2 large chain _F5B8W8_LUPAN	(8.00 ± 0.4) × 10^3^	(6.76 ± 0.1) × 10^3^	(1.20 ± 0.06) × 10^4^	(1.72 ± 0.05) × 10^4^	46.51	39.30	69.77

**Table 2:** Amount of γ-conglutin found in lupin-based pasta.

Sample	Amount of γ-Conglutin (mg/g)	RSD%
A	2.25	12.2
D	2.16	3.5
F	0.57	11.3

## Supplementary Materials

The following are available online at https://www.mdpi.com/2304-8158/9/8/1083/s1, Table S1: HPLC-Chip-ESI-MS/MS based identification of peptides per proteins, respectively, identified in all the 6 analyzed samples. The untargeted MS parameters used are reported in Supplementary Materials.

## Abbreviations

ACN, acetonitrile; BSA, bovine serum albumin; DTT, 1,4-dithiothreitol; DW, durum wheat flour (DW); FDR, false discovery rate; HCA, Hierarchical clustering analysis; HCl, hydrochloric acid (HCl); HPLC-ESI-MS/MS, High-performance liquid chromatography electrospray ionization tandem mass spectrometry; iBAQ, intensity-based absolute quantification; IAM, 2-iodoacetamide; MRM, multiple reactions monitoring; LOD, limit of detection; LOQ limit of quantification; LK, lupin kernel; MW, molecular weight; RDS%, standard deviation percentage; SDS-PAGE, sodium dodecyl sulphate-polyacrylamide gel electrophoresis; TIC, total ion chromatogram; Tris-HCl, tris(hydroxymethyl)aminomethane.
